# The biology and pathophysiology of the proatherogenic metalloprotease ADAMTS7

**DOI:** 10.1098/rsob.250111

**Published:** 2026-04-01

**Authors:** Shiwali Goyal, James Muniu, Mohd Akbar Bhat, Eli Oldham, Derrick W. Pruitt, Luis Frias Perez, Frida Z. Miranda, Joshua Muia

**Affiliations:** ^1^ National Institute of Health, National Eye Institute, Bethesda, MD, USA; ^2^ Oklahoma State University College of Osteopathic Medicine, Tulsa, OK, USA; ^3^ Department of Ophthalmology, Georgetown University Medical Center, Washington, DC, USA; ^4^ Department of Biochemistry and Microbiology, Oklahoma State University Center for Health Sciences, Tulsa, OK, USA; ^5^ Department of Biochemistry, Medical College of Wisconsin, Milwaukee, WI, USA; ^6^ Thrombosis and Hemostasis Program, Versiti Blood Research Institute, Milwaukee, WI, USA

**Keywords:** ADAMTS7, ADAMTS metalloproteases, atherosclerosis, coronary artery disease, GWAS

## Abstract

The ADAMTS (a disintegrin and metalloproteinase with thrombospondin type-1 motifs) metalloprotease family comprises 19 members, and they play critical roles in development, angiogenesis and coagulation. These enzymes are also pivotal in the turnover of extracellular matrix proteins in various tissues, and their dysregulation has been linked to diseases such as arthritis, atherosclerosis, cancer and inflammation. These secreted zinc metalloproteases are composed of multiple domains and are localized to the extracellular matrix and present in circulation. The metalloprotease member 7 of this family or ADAMTS7 was cloned in 2004. However, in 2011, it gained significant attention when its gene locus was associated with an increased risk of atherosclerosis and coronary artery disease. Despite its strong association with human diseases, the biological functions, substrate targets and processing of ADAMTS7 remain poorly understood, limiting our comprehension of its roles in both health and disease. This review highlights the structural organization of ADAMTS7, its expression and regulation, known substrates, and its involvement in various pathophysiological processes. Additionally, perspectives on future research directions and priorities are presented. ADAMTS7 remains a compelling target for drug and biomarker discovery, though much about this metalloprotease remains to be uncovered.

## 1. Introduction to ADAMTS7 and its ADAMTS metalloprotease family

The ADAMTS (a disintegrin and metalloprotease with thrombospondin type-1 motifs) protein family has been implicated in many biological processes such as blood coagulation, cell adhesion and migration, proteolysis and extracellular remodelling [1,2] (table 1). In 1997, Kuno and colleagues [3] were the first to identify an ADAMTS protease in mice. Since then, more similar metalloproteases have been found in other mammals and invertebrates, including *Caenorhabditis elegans* [4–6]. Further research revealed that of the 26 ADAMTS superfamily genes known in mice and humans, 19 encode zinc metalloproteinases, constituting the ADAMTS protease family (ADAMTS1 through ADAMTS20). It was later discovered that *ADAMTS5* and *ADAMTS11* are the same genes; hence, *ADAMTS11* is no longer used [1,7]. The identification of genes encoding the seven ADAMTS-like proteins (ADAMTSL1 through ADAMTSL6 and PAPLN) completed the superfamily’s genetic cataloguing. These proteins possess no catalytic activity; however, they are thought to regulate the activities of the ADAMTSs as well as being involved in various human pathologies [8–14]. ADAMTS7 is the ADAMTS family member 7, according to discovery and nomenclature, and its functions and pathophysiology in inflammation, arthritis, and atherosclerosis are actively investigated.

**Table 1.** ADAMTS metalloproteases, substrates and pathophysiology. ADAMTS proteases are associated with various clinical manifestations, including arthritis, cancer, blood coagulation and atherosclerosis. vWFCP: von Willebrand factor cleaving protease; COMP: cartilage oligomeric matrix protein.

gene name	chromosomal location	substrate	pathological association
*ADAMTS1*	21q21	aggrecanase/proteoglycanase	cancer, atherosclerosis, fibrosis, antiangiogenesis, ovarian function and stress
*ADAMTS2*	5q35	procollagen N-propeptidase	Ehlers–Danlos syndrome, mesothelioma and placenta development
*ADAMTS3*	4q21	procollagen N-propeptidase	dermatosparaxis, osteoarthritis and lymphangiogenesis
*ADAMTS4*	1q23	aggrecanase/proteoglycanase	glioma, atherosclerosis, arthritis and tendinopathy
*ADAMTS5*	21q21	aggrecanase/proteoglycanase	arthritis, cancer
*ADAMTS6*	5q12	unknown	tissue repair
*ADAMTS7*	5q24	COMP proteinases	arthritis, atherosclerosis and kidney damage
*ADAMTS8*	11q24	aggrecanase/proteoglycanase	cancer, atherosclerosis, arthritis and antiangiogenesis
*ADAMTS9*	3p14	aggrecanase/proteoglycanase	cancer, atherosclerosis, arthritis and antiangiogenesis
*ADAMTS10*	19p13	unknown	Weill-Marchesani syndrome
*ADAMTS12*	5p13	COMP proteinases	arthritis, cancer and normal inflammatory response
*ADAMTS13*	9q34	vWFCP	thrombotic thrombocytopenic purpura
*ADAMTS14*	10q22	procollagen N-propeptidase	fibrosis, osteoarthritis, tendon disorders and sclerosis
*ADAMTS15*	11q24	aggrecanase/proteoglycanasce	cancer, follicle rupture, myogenesis and spinal injury
*ADAMTS16*	5p15	unknown	cancer, cryptorchidism and premature ovarian failure
*ADAMTS17*	15q26	unknown	Weill–Marchesani-like syndrome, short-stature and paediatric stroke
*ADAMTS18*	16q23	unknown	ocular disease, cancer, stroke and bone disorders
*ADAMTS19*	5q23	unknown	premature ovarian failure, osteosarcomas
*ADAMTS20*	2q12	aggrecanase/proteoglycanase	melanocyte, differentiation, pigmentation and apoptosis

### 1.1. ADAMTS7 significance and overview

Major ADAMTS7 research milestones in the past 20 years have been summarized chronologically in figure 1. ADAMTS7 has garnered a lot of attention due to its association with atherosclerosis and coronary artery disease (CAD) [15,16]. Many human studies have supported a strong link between ADAMTS7 and atherosclerosis [17–23]. While the clinical/genotype data underpin the role of ADAMTS7 in atherosclerosis progression, its functional role in atherosclerosis and CAD remains incompletely understood [24]. The present review highlights the current knowledge of ADAMTS7, including its structure, function, biology and pathophysiology.

**Figure 1.**
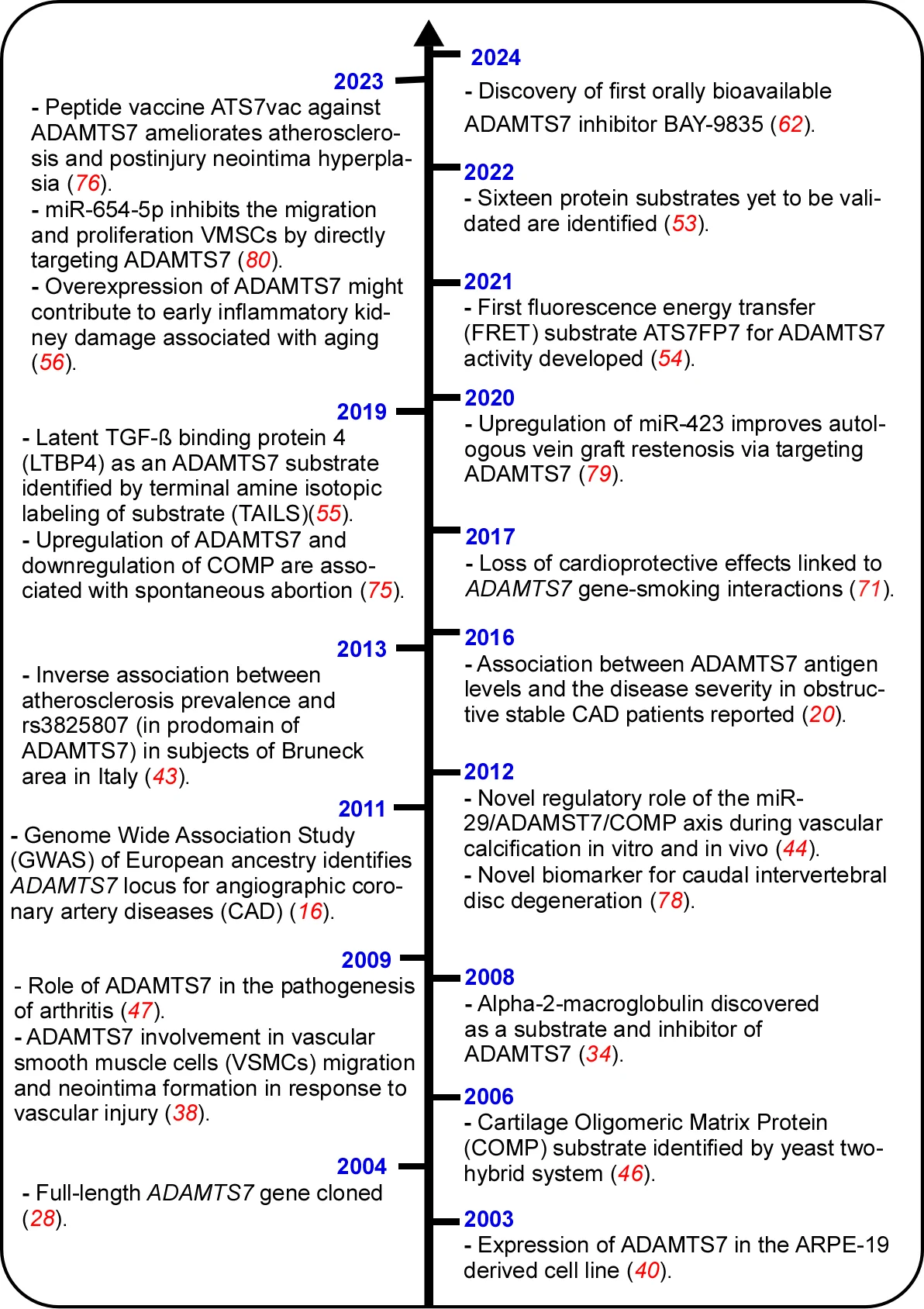
A timeline of significant studies of the metalloprotease ADAMTS7. The full-length ADAMTS7 was cloned approximately 20 years ago, and despite being associated with several disorders, the mechanisms by which it drives the pathophysiology in these diseases are not well understood. ADAMTS7 substrate identification and validation are areas of research that remain critical to unravel the biology and pathophysiology of the metalloprotease. The cited work is in red font.

Large-scale GWAS first implicated ADAMTS7 in CAD. Lead variants at this locus, commonly rs3825807, rs1994016 and rs4380028, show reproducible associations across cohorts [16,25]. Subsequent work reported a gene–environment interaction at rs7178051, where the cardioprotective allele conferred less benefit in smokers, consistent with smoke-induced upregulation of ADAMTS7 in vascular cells [26]. These findings established ADAMTS7 as a CAD locus.

### 1.2. ADAMTS7 structure and discovery

The ADAMTS7 metalloprotease in human is encoded by the *ADAMTS7* gene, which spans 52.3 kb of the human genome, has 24 exons, and maps to chromosome 15q24. ADAMTS7 was first identified and cloned in 1999 [27], encoding a secreted metalloprotease with the typical ADAMTS architecture, including two C-terminal thrombospondin type-1 (TSR) motifs. In 2004, a longer splice isoform of the same gene—ADAMTS7B—was described; it features an extended C-terminus with additional TSRs (and a mucin-rich segment) relative to the canonical isoform [28].

Like most of the ADAMTS metalloproteases [29,30], ADAMTS7 is a zymogen with a signal peptide, a prodomain, a catalytic metalloproteinase domain, a disintegrin-like domain, a central TSP-1 repeat, a cysteine-rich domain and seven additional TSP-1 repeats spaced apart by two cysteine-free spacer domains. As other selected ADAMTS proteases (ADAMTS2, -3, -6, -10, -12, -16 and -19) [6], ADAMTS7 has a C-terminal PLAC (protease and lacunin) domain with six cysteines in the usual configuration. The second spacer domain of ADAMTS7 is a mucin-like domain (figure 2) [28,31,32]. ADAMTS7 is synthesized as a prepro-enzyme and becomes active after removal of its N-terminal prodomain by a furin-like proprotein convertase in the trans-Golgi network; the mature enzyme can then undergo further extracellular processing [33]. Secreted ADAMTS7 might bind to protein substrates such as α2-macroglobulin (α2M) [34], connective tissue growth factor (CTGF) [35], granulin-epithelin precursor (GEP) [36] and cartilage oligomeric matrix protein (COMP) [37]. ADAMTS7 degrades the extracellular matrix of vessel walls, most notably by cleaving COMP [28,31,38]. Interestingly, both α2-macroglobulin and GEP prevent ADAMTS7 cleavage of COMP and protect it from degradation *in vitro* [39].

**Figure 2.**
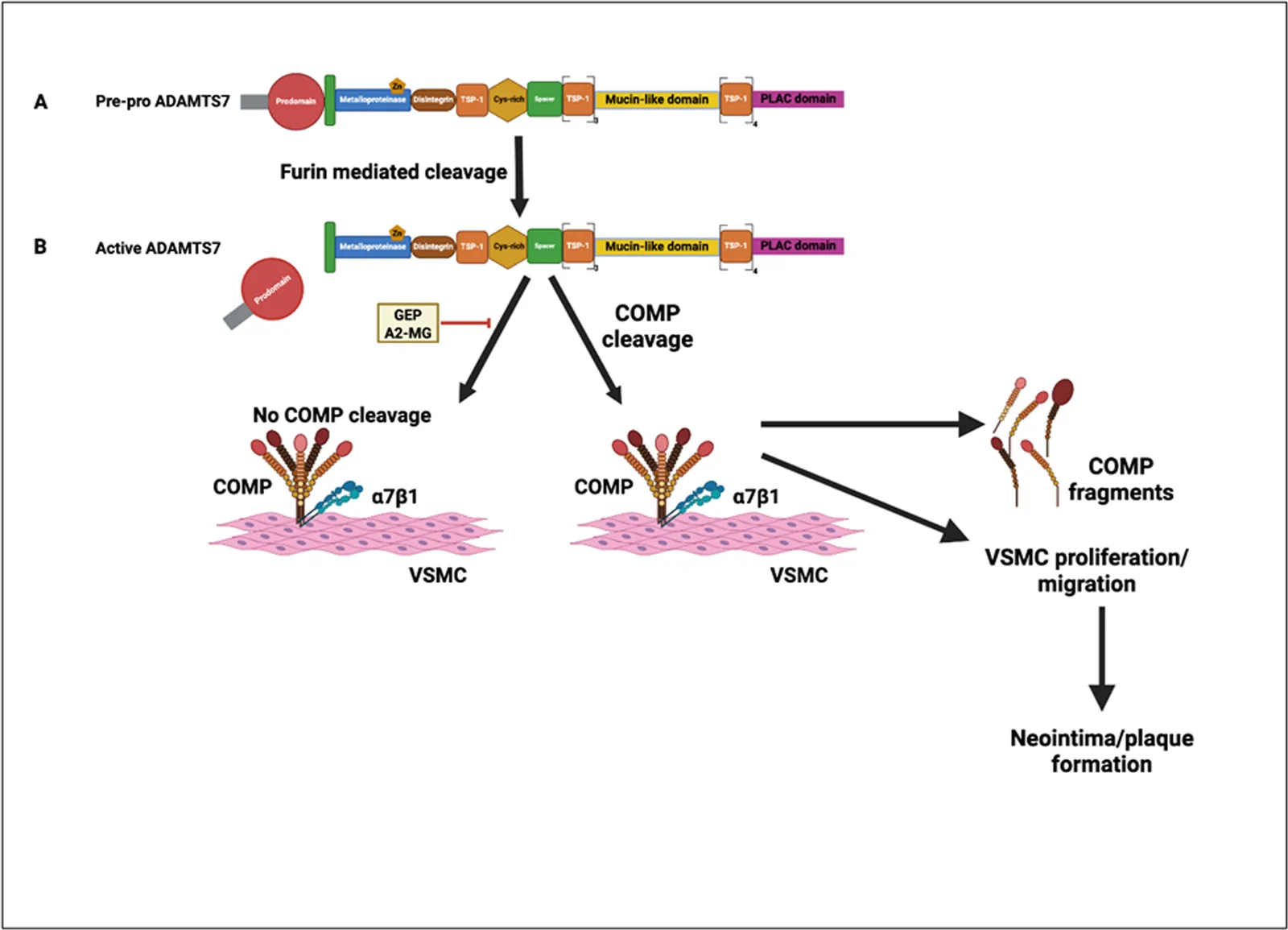
The structure of ADAMTS7 and its putative role in vascular smooth muscle cell (VSMC), proliferation and migration. COMP binds to the α7β1 integrin and is cleaved by ADAMTS7 to form a complex with VSMCs. GEP: granulin-epithelin precursor; A2M: α2-macroglobulin.

### 1.3. ADAMTS7 expression and regulation

ADAMTS7 protein expression was initially studied in ARPE-19 cells [40,41]. TNFα induced ADAMTS7 expression in osteoarthritis (OA) rat models [40]. Additionally, the expression and detection of ADAMTS7 have been studied using animal models [41,42], *in vitro* cell-based assays [43] and patient specimens [24]. *Adamts7* mRNA is highly expressed in the heart of C57BL/6 J mice, with moderate levels in the brain, lung and duodenum, and basal levels in the liver, spleen and thyroid [41]. *In vitro*, ADAMTS7 is produced and secreted by macrophages; however, direct tests of ADAMTS7’s effect on efferocytosis remain limited, and a mechanistic link has not been established [31,37,39]. In humans, ADAMTS7 expression has been observed in coronary arteries and carotid atherosclerotic lesions where higher levels correlate with plaque instability and cardiovascular risk [17,41,43].

Cytokines, growth factors and free radicals can regulate the expression of ADAMTS7 [36]. Proinflammatory cytokines, tumour necrosis factor-alpha (TNF-α) and interleukin-1 beta (IL-1β), and platelet-derived growth factor two B subunits (PDGF-BB) induce *ADAMTS7* mRNA and protein levels in the cultured vascular smooth muscle cells (VSMCs) [34,38]. Promoter analyses identified NFκB and AP-1 binding sites, confirmed by ChIP assays and inhibition of these pathways markedly reduced TNF-induced ADAMTS7 expression [31,38].

MicroRNAs also contribute to regulation. miR-29a/b directly targets the 3′UTR of *ADAMTS7*, with mimics decreasing and inhibitors increasing ADAMTS7 expression in VSMCs [31,38,44]. Collectively, ADAMTS7 protein expression is tightly regulated by inflammatory signalling, oxidative stress and miRNAs, and its dysregulation contributes to vascular remodelling and atherosclerosis.

## 2. The known ADAMTS7 substrates

Several putative ADAMTS7 substrates have been identified, including COMP or thrombospondin-5 (TSP-5), GEP or progranulin (PGRN) and α2M [45]. COMP can be cleaved by ADAMTS7; GEP interferes with ADAMTS7-mediated COMP cleavage by generating complex protein–protein networks; and α2M prevents ADAMTS7-mediated COMP cleavage [31,34].

### 2.1. COMP: the most characterized ADAMTS7 substrate

Cartilage oligomeric matrix protein is a 524 kDa pentameric glycoprotein found in cartilage and arteries that was identified as an ADAMTS7 substrate through protein–protein interactions using yeast two-hybrid (Y2H) screening [46]. Immunoprecipitation and degradation experiments confirmed and validated the interactions between COMP and ADAMTS7 [47,48].

In the vascular system, COMP plays an important role in vascular smooth muscle cell biology. COMP interacts with the α7β1 integrin to keep VSMCs contractile and quiescent state. Cleavage of COMP by ADAMTS7 disrupts this interaction, shifting VSMCs towards a migratory phenotype and promoting neointima formation after artery injury [38,49] (figure 2). Additionally, COMP inhibits vascular calcification by binding bone morphogenetic protein-2 (BMP-2), thereby blocking osteogenic signalling. ADAMTS7-mediated COMP degradation removes this inhibitory effect, contributing to vascular calcification [50].

### 2.2. Granulin-epithelin precursor (GEP)

Granulin-epithelin precursor is an 88 kDa (593-aa) growth factor released by cells. It is also known as progranulin, proepithelin, or plasma cell-derived growth factor [28,51]. GEP interferes with ADAMTS7-mediated COMP cleavage by forming complex protein–protein interactions. In an ApoE knockout mouse model, loss of progranulin aggravated atherosclerotic lesions, supporting its protective role in vascular biology [52]. Thus, GEP serves as a modulator of ADAMTS7 activity and contributes to vascular remodelling.

### 2.3. α2-macroglobulin (α2M)

α2-macroglobulin is a high molecular weight (720 kDa) homotetrameric glycoprotein found in plasma [34]. ADAMTS7 cleaves α2M *in vitro*, but importantly, α2M is the first identified endogenous inhibitor of ADAMTS7, preventing COMP degradation [34]. This inhibitory effect provides a natural mechanism of regulating ADAMTS7 activity in vascular tissues.

## 3. Recent advances in ADAMTS7 substrate identification

There has been concerted efforts in recent times to elucidate the full spectrum of ADAMTS7 interaction partners and substrate targets [24,53–56]. Recent investigations continue to substantiate the proteolytic role of ADAMTS7 in cardiovascular and inflammatory conditions with mechanistic implications in blood vessel elastic fibre production [57], complement-mediated disorders [58] and collagen content in arteries [59]. Methodologies for high-throughput screening of ADAMTS7 inhibitors [60] and potential substrates [61] have also been reported.

Colige *et al.* [55] used the terminal amine isotopic labelling of substrates (TAILS) technique to identify latent TGF-β binding protein 4 (LTBP4) as a candidate ADAMTS7 substrate. LTBP4, a component of microfibrils/elastic fibres in the lung and major blood vessels, is co-expressed with ADAMTS7 in the heart and interacts with fibrillin-1, fibulin-4 and fibulin-5, which are essential for elastic fibre assembly. LTBP4 proteolysis by ADAMTS7 may promote elastic fibre remodelling, although its *in vivo* role and contribution to atherosclerosis remain to be established. In a complementary TAILS study, MacDonald *et al.* [53] analysed wild-type and inactive catalytic mutant (E373Q) murine Adamts7 in human coronary artery smooth muscle and endothelial cells, identifying 24 cleavage sites across 16 potential substrates (including AEBP1, COL18A1, EFEMP1, FBN1, FLNA, FN1, HSPG2, IGFBP7, LOX, LOXL2, LTBP1, LTBP3, MAP4, NID2, SERBP1 and SRGN). Although these candidate substrates await validation, both studies underscore the utility of TAILS in uncovering novel ADAMTS7 substrates and highlight the need for further functional characterization.

Santamaria *et al.* [54] described the fluorescence resonance energy transfer (FRET) substrate, ATS7FP7, for ADAMTS7. ATS7FP7 was used to measure the inhibition constants of TIMP-4, an endogenous ADAMTS7 inhibitor, and two hydroxamate-based zinc chelating inhibitors. These inhibition constants were consistent with the IC_50_ values obtained using SDS-PAGE experiments; hence, the authors confirmed that the ATS7FP7 fluorogenic substrate may be suitable for high-throughput screening of ADAMTS7 inhibitors. Recently, Meibom *et al.* identified an ADAMTS7 catalytic site target inhibitor. Using a dual ADAMTS4/ADAMTS5 inhibitor as a template, the authors developed BAY-9835, the first orally bioavailable ADAMTS7 inhibitor, with improved selectivity and potency [62]. Efforts to enhance the BAY-9835 selectivity to ADAMTS7 and the related metalloprotease ADAMTS12 are ongoing.

Ma *et al.* [56] documented complement factor H (CFH), an essential inhibitor of alternate complement system pathways in the kidneys of a lupus mouse model, as a novel ADAMTS7 substrate. Furthermore, ADAMTS7 overexpression was associated with decreased CFH in both lupus mice and humans. CFH dysfunction results in complement activation that may lead to complement-mediated diseases. ADAMTS7 is linked to the CFH complement control protein (CCP) 1–4 domain and degrades the CCP 1–7 domain through recurred cleavages. These protective effects of ADAMTS7 deletion against complement-mediated kidney damage *in vivo* were undermined by adeno-associated virus-mediated CFH silencing [56].

In a recent investigation by Sharifi *et al.* [24], the metalloproteinases-1 (TIMP-1) tissue inhibitor was identified as a target substrate of ADAMTS7. TIMP-1 is pivotal in regulating extracellular matrix remodelling by inhibiting matrix metalloprotease 9 (MMP-9), thus preventing collagen degradation within arteries. ADAMTS7, however, reduces the stability of TIMP-1, thereby relieving the inhibition of MMP9 and leading to decreased collagen content in arteries. The researchers suggested that disrupting the interaction between ADAMTS7 and TIMP-1 could potentially enhance collagen content and plaque stability, ultimately reducing atherosclerosis-related events.

Overall, the substrate specificity of ADAMTS7 is still poorly characterized. A more comprehensive analysis of its substrate repertoire and cleavage sites is warranted. Understanding the functional effects of proteolysis by ADAMTS7 requires knowledge of its cleavage sites. This knowledge may also guide the development of (therapeutic) inhibitors that gain selectivity and efficacy by imitating substrate residues flanking the cleavage site.

## 4. Pathophysiology of diseases associated with ADAMTS7 metalloprotease

Due to the broad spectrum of identified putative and candidate ADAMTS7 substrates, the implication of ADAMTS7 proteolytic activity in diseased states is of therapeutic interest.

### 4.1. ADAMTS7 and bone and joint disease

For many years, research into the physiological activities of ADAMTS7 has focused chiefly on its relationship with the aetiology of arthritis [48,63] and disc disease [64,65]. This was due to the observation that COMP was predominantly found in the cartilage extracellular matrix, accounting for 1% of the wet weight of cartilage. More importantly, increased fragments of COMP were detected in the serum and synovial fluid of patients with rheumatoid arthritis and OA [31,46]. ADAMTS7 overexpression was also seen in the cartilage and synovium of rheumatoid arthritis patients. Additionally, COMP cleavage *in vitro* by recombinant ADAMTS7 revealed the identical COMP cleaved fragments reported in the serum and synovial fluid of rheumatoid arthritis and OA patients [46]. Several studies have demonstrated that under physiological conditions, normal expression levels of ADAMTS7 maintain the baseline levels of COMP cleavage in chondrocytes for chondrogenesis. Excess COMP cleavage, conversely, causes degenerative and inflammatory disorders of the joints and intervertebral discs (IVD) in pathological situations when *ADAMTS7* gene expression is upregulated [31,46,66,67].

### 4.2. ADAMTS7 and cardiovascular disease

In 2009, researchers discovered that ADAMTS7 has a functional aetiology in vascular diseases [38]. A rat balloon injury model found that ADAMTS7 increased VSMC migration by cleaving COMP and accelerated neointima formation following vascular mechanical damage [38]. Neointima development is thought to be an aggressive reaction to vascular damage, such as that found after coronary stent placement [32]. Wang *et al.* [38] discovered an initial drop in ADAMTS7 protein levels in the arterial wall during the first 24 h following vascular damage, followed by a rise in ADAMTS7 levels up to 2 weeks later, with peak expression after 1 week. ADAMTS7 preferentially accumulated in the neointima and colocalized with VSMCs [38]. Overexpression of ADAMTS7 in VSMCs via adenovirus infection increased VSMC migration/invasion *in vitro* and neointima formation *in vivo*. Whereas, small interfering RNA (siRNA) suppression of ADAMTS7 retarded VSMC migration *in vitro* and improved neointima formation in response to injury *in vivo*. Furthermore, it was showed that following mechanical damage to the rat carotid artery, COMP fragments increased in conjunction with ADAMTS7 levels [38]. Therefore, ADAMTS7 may influence VSMC migration by cleaving its primary substrate, COMP. Similarly, overexpression of ADAMTS7 decreased full-length COMP and increased fragments of COMP, whereas inhibition of ADAMTS7 had the reverse effect. Furthermore, replenishment of COMP through adenovirus infection reduced ADAMTS7-mediated VSMC migration [38]. In an *Adamts7* mouse knockout model, the loss of Adamts7 resulted in impaired neointima development following carotid artery damage produced by ligation [68]. VSMC migration is a crucial phase in atherogenesis, and neointima development is critical in restenosis following angioplasty. These findings establish ADAMTS7 as a key factor in the pathophysiology of cardiovacular disease [68].

Genome-wide association studies (GWAS) have now validated the role of ADAMTS7 in cardiovascular diseases [16,69]. Single nucleotide polymorphisms (SNPs: rs3825807, rs1994016, rs4380028, rs79265682 and rs28455815) found in the *ADAMTS7* gene locus were reported to be strongly linked with CAD [16,69]. Petrovič *et al.* [70] reported a statistically significant correlation between rs3825807 (c.640G > A) and myocardial infarction within a cohort of Slovenian patients diagnosed with type 2 diabetes mellitus. The authors also reported that individuals with the AA genotype exhibited a greater prevalence of myocardial infarction compared to the control group in both recessive (odds ratio (OR) 1.647; confidence interval (CI) 1.120–2.407; *p* = 0.011) and co-dominant (OR 2.153; CI 1.215–3.968; *p* = 0.011) genetic models. A meta-analysis of the studies indicated that the *ADAMTS7* SNP rs3825807 risk allele A had the strongest association with CAD, followed by rs4380028 (C versus T allele) and rs1994016 (C versus T allele) [23]. However, it is unknown if any of these GWAS SNPs or other SNPs in linkage disequilibrium (LD) have a functional effect on ADAMTS7 expression/activity and influence the biological processes connected to CAD. Functional investigations of these *ADAMTS7* gene variations will reveal plausible mechanisms driving these relationships.

Saleheen *et al.* [71] explored the interaction between smoking behaviour and genetic variants linked to coronary heart disease (CHD). Analysing data from over 140 000 individuals, authors identified a novel gene-smoking interaction at the rs7178051 variant near the *ADAMTS7* gene. The T allele of rs7178051 was associated with a 12% lower risk of CHD in non-smokers but only a 5% reduction in smokers, indicating a 60% loss of CHD protection in those who smoked. The study suggests that increased ADAMTS7 expression in vascular cells, triggered by smoking, may explain this diminished protection.

### 4.3. Potential role of ADAMTS7 in cancer

Many ADAMTS family members are dysregulated or overexpressed in various human cell malignancies [72]. ADAMTS7 was found in the urine of individuals with prostate, brain, bladder, breast and liver carcinomas in one investigation [48]. Further investigation revealed that ADAMTS7 is present in the urine of patients with breast, bladder and prostate cancer but not in control urine, but whether ADAMTS7 plays a role in the development or invasion of these malignancies remains unknown [73].

### 4.4. ADAMTS7 in the pathogenesis and progression of elderly kidney damage

Gao *et al.* investigated the role of ADAMTS1, -4, -7 and MMP-9 in inflammatory kidney damage in elderly mice after Angiotensin II (Ang II) administration [74]. The results showed that Ang II treatment caused hypertension and kidney damage, with a significant increase in ADAMTS7 expression at both the mRNA and protein levels. In contrast, norepinephrine treatment did not induce kidney damage or affect ADAMTS7 expression. These findings suggest that overexpression of ADAMTS7, induced by Ang II, may contribute to early inflammatory kidney damage in ageing.

### 4.5. Role of ADAMTS7 and COMP expression in spontaneous abortion

Mu *et al.* investigated the role of ADAMTS7 in spontaneous abortion (SA) using human and mouse decidual tissues [75]. In mice with lipopolysaccharide (LPS)-induced abortion, Adamts7 expression was upregulated, and COMP expression was downregulated. Similar findings were observed in the decidual tissue of SA patients. A negative correlation was found between Adamts7 and COMP expression in mice (*r* = −0.936) and humans (*r* = −0.836). These results suggest that ADAMTS7 upregulation and COMP downregulation in decidual tissue may contribute to SA, offering insights into its pathogenesis and potential clinical implications for improving pregnancy outcomes.

## 5. ADAMTS7 as a therapeutic target and biomarker

Experiments by Ma *et al.* [76] demonstrated that disruption of Adamts7 or its catalytic activity reduces the atherosclerosis burden in murine models. The authors developed a vaccine, ATS7vac, against Adamts7 using three epitopes of its secondary structure at the catalytic domain and the C-terminal TSP-1 repeats. The fact that ATS7vac reduced aortic plaques by about 50% shows that targeting ADAMTS7 in atherosclerosis may become a promising strategy for addressing the remaining risk of atherosclerosis after lowering cholesterol levels. In the same study, Ma *et al.* evaluated the possibility of inhibiting ADAMTS7 to diminish in-stent restenosis with ATS7vac in a swine restenosis model. As predicted by the mouse models, ATS7vac decreased neointima development following stent insertion in swine coronary arteries. Identifying novel ADAMTS7 substrates is an important future direction to unravel its function and thus, therapeutics in CAD.

Further, Kessler *et al.* [77] followed up with the limited understanding of downstream mechanisms beyond ADAMTS7-mediated SVEP1 (sushi, von Willebrand factor type A, EGF and pentraxin domain containing 1) degradation by questioning the reproducibility of vaccination findings with other extracellular matrix proteases. Though Ma *et al.* [76] found no safety issues with the ATS7vac, robust preclinical testing is needed before patient trials. Trials in atherosclerosis patients require large sample sizes (>2000 high-risk patients) to detect significant reductions (approx. 15%) in major cardiovascular events. Similarly, restenosis studies face challenges due to reduced incidences with drug-eluting stents, making the detection of clinical benefits difficult.

Yu and Zhu investigated the role of ADAMTS7 and ADAMTS12 in intervertebral discs degeneration [78]. Using rat caudal discs under compression, authors observed reduced cell viability, collagen II and aggrecan, alongside increased ADAMTS7, ADAMTS12 and COMP fragments. Although COMP initially rose, it was ultimately degraded by ADAMTS7 and ADAMTS12, exacerbating matrix degradation. These findings suggest that ADAMTS7, ADAMTS12 and COMP are potential markers of disc degeneration.

Ren *et al.* [79] reported that the miR-423/ADAMTS7 axis regulates vein graft restenosis in coronary artery bypass grafting (CABG). They found that miR-423 suppresses ADAMTS7, reducing smooth muscle cell proliferation and enhancing endothelial cell function, suggesting its therapeutic potential for vein graft failure. Recently, Li *et al.* [80] explored the role of microRNA-654-5p (miR-654-5p) in CAD by examining its effects on VSMCs. miR-654-5p was found to be downregulated in CAD patients and PDGF-BB-stimulated VSMCs. Transfecting VSMCs with miR-654-5p mimic inhibited their migration and proliferation, while a miR-654-5p inhibitor promoted these processes. ADAMTS7 was identified as a direct target of miR-654-5p, and its overexpression counteracted the inhibitory effects of miR-654-5p. These findings suggest miR-654-5p as a potential therapeutic target for CAD by regulating VSMC migration and proliferation through ADAMTS7.

Although novel therapeutics targeting ADAMTS7 mechanistic pathways are in the initial stages of development, a more thorough understanding of ADAMTS7 and its functional activity is necessary. Emerging research linking ADAMTS7 to cardiovascular disease substantiates this need further to elucidate the role of ADAMTS7 in atherosclerosis and CAD. Cardiovascular disease is the global leading cause of death, and as a multifactorial illness, selective therapeutics are challenging to design. A more comprehensive understanding of ADAMTS7 proteolytic activity, substrates and cleavage sites will be a roadmap for a new class of CAD therapeutics.

## 6. ADAMTS7 future outlook and research priorities

A central priority is the rigorous conversion of candidate interactors into bona fide ADAMTS7 substrates with defined mechanisms of action. Beyond COMP, proteins highlighted by discovery proteomics and targeted assays should remain ‘candidates’ until supported by an orthogonal evidence stack that: (i) demonstrates direct proteolysis by purified ADAMTS7; (ii) eliminates cleavage with a catalytically inactive enzyme and/or selective inhibitors; (iii) maps N- and C-terminal neo-junctions at amino-acid resolution; and (iv) links proteolysis to loss- and gain-of-function phenotypes in relevant cellular and tissue contexts [53]. *In vivo* work should pair substrate verification with cardiovascular outcome changes in vascular ECM composition, VSMC contractile→migratory transitions, arterial collagen content and plaque stability, such as, using temporal designs that place ADAMTS7 activity upstream of these readouts [24,81]. Where feasible, substrate-specific rescue (re-expression of cleavage-resistant mutants) can provide causal inference between proteolysis and phenotype.

In parallel, enabling reagents are needed to define where and when ADAMTS7 acts: selective chemical probes and inhibitors for activity tracking and perturbation; high-specificity antibodies for localization and pharmacodynamic assays; and genetic tools (conditional/inducible alleles, catalysis-dead knock-ins) to resolve tissue- and stage-specific functions. These should be integrated into pathways already implicated in ADAMTS7 control-pro-inflammatory signalling, oxidative stress and miRNA regulation to distinguish pathogenic from homeostatic roles [24,81]. Finally, areas with early signals merit targeted exploration, including cross-talk with other metalloproteases [25], roles in wound repair [42] and links to inflammation and immunity [81], with the goal of refining indication selection, biomarker strategies and combination approaches for ADAMTS7-directed therapies.

## 7. Conclusions

ADAMTS7 has emerged as a potential player in various physiological and pathological processes, particularly in cardiovascular diseases, OA and other conditions influenced by extracellular matrix dynamics [25,26,36,42,82]. The ongoing research highlights its potential as a therapeutic target, yet significant gaps remain in our understanding of its physiologic substrates and molecular mechanisms. Future studies will be essential to validate proposed substrates, elucidate their role in disease pathophysiology and develop robust research tools. By addressing these challenges, we can advance understanding of ADAMTS7 in both basic and translational research, paving the way for innovative therapeutic strategies that leverage its unique biological functions.

## Data Availability

This article has no additional data.
